# Traumatic Brain Injury Outcome Associations With Computed Tomography and Glasgow Coma Scale Score Interactions: A Retrospective Study

**DOI:** 10.7759/cureus.53781

**Published:** 2024-02-07

**Authors:** C. Michael Dunham, Gregory S Huang, Kene T Ugokwe, Brian P Brocker

**Affiliations:** 1 Trauma, Critical Care, and General Surgery Services, St Elizabeth Youngstown Hospital, Youngstown, USA; 2 Department of Neurosurgery, St Elizabeth Youngstown Hospital, Youngstown, USA

**Keywords:** brain ct scan, glasgow outcome scale, glasgow coma scale, neurotrauma, decompressive craniotomy, traumatic brain injury

## Abstract

Background

Numerous investigators have shown that early postinjury Glasgow Coma Scale (GCS) values are associated with later clinical outcomes in patients with traumatic brain injury (TBI), in-hospital mortality, and post-hospital discharge Glasgow Outcome Scale (GOS) results. Following TBI, early GCS, and brain computed tomography (CT) scores have been associated with clinical outcomes. However, only one previous study combined GCS scores with CT scan results and demonstrated an interaction with in-hospital mortality and GOS results. We aimed to determine if interactive GCS and CT findings would be associated with outcomes better than GCS and CT findings alone.

Methodology

Our study included TBI patients who had GCS scores of 3-12 and required mechanical ventilation for ≥five days. The GCS deficit was determined as 15 minus the GCS score. The mass effect CT score was calculated as lateral ventricular compression plus basal cistern compression plus midline shift. Each value was 1 for present. A prognostic CT score was the mass effect score plus subarachnoid hemorrhage (2 if present).The CT-GCS deficit score was the sum of the GCS deficit and the prognostic CT score.

Results

One hundred and twelve consecutive TBI patients met the inclusion criteria. Patients with surgical decompression had a lower GCS score (6.0±3.0) than those without (7.7±3.3; Cohen d=0.54). Patients with surgical decompression had a higher mass effect CT score (2.8±0.5) than those without (1.7±1.0; Cohen d=1.4). The GCS deficit was greater in patients not following commands at hospital discharge (9.6±2.6) than in those following commands (6.8±3.2; Cohen d=0.96). The prognostic CT score was greater in patients not following commands at hospital discharge (3.7±1.2) than in those following commands (3.1±1.1; Cohen d=0.52). The CT-GCS deficit score was greater in patients not following commands at hospital discharge (13.3±3.2) than in those following commands (9.9±3.2; Cohen d=1.06). Logistic regression stepwise analysis showed that the failure to follow commands at hospital discharge was associated with the CT-GCS deficit score but not with the GCS deficit. The GCS deficit was greater in patients not following commands at three months (9.7±2.8) than in those following commands (7.4±3.2; Cohen d=0.78). The CT-GCS deficit score was greater in patients not following commands at three months (13.6±3.1) than in those following commands (10.5±3.4; Cohen d=0.94). Logistic regression stepwise analysis showed that failure to follow commands at three months was associated with the CT-GCS deficit score but not with the GCS deficit. The proportion not following commands at three months was greater with a GCS deficit of 9-12 (50.9%) than with a GCS deficit of 3-8 (21.1%; odds ratio=3.9; risk ratio=2.1). The proportion of not following commands at three months was greater with a CT-GCS deficit score of 13-17 (56.0%) than with a CT-GCS deficit score of 4-12 (18.3%; OR=5.7; RR=3.1).

Conclusion

The mass effect CT score had a substantially better association with the need for surgical decompression than did the GCS score. The degree of association for not following commands at hospital discharge and three months was greater with the CT-GCS deficit score than with the GCS deficit. These observations support the notion that a mass effect and subarachnoid hemorrhage composite CT score can interact with the GCS score to better prognosticate TBI outcomes than the GCS score alone.

## Introduction

Numerous investigators have shown that early postinjury Glasgow Coma Scale (GCS) values are associated with later clinical outcomes in patients with traumatic brain injury (TBI). Research also demonstrates that early postinjury GCS scores are associated with in-hospital mortality [1-3]. Investigations also show that early postinjury GCS scores are associated with post-hospital discharge Glasgow Outcome Scale (GOS) results [1-7].

Several studies have also shown that early TBI computed tomography (CT) scores are associated with subsequent clinical outcomes. Specifically, numerous investigations have shown that early CT score findings have associations with in-hospital mortality. Using Rotterdam and Marshall CT scores in 1,035 patients with TBI, Asim et al. showed that increased scores were associated with in-hospital mortality [8]. In a study of patients from the United States, Elkbuli et al. analyzed Rotterdam and Marshall CT scores and demonstrated that higher score values were associated with hospital mortality [9]. Further, an Indian investigation of 127 patients with TBI that used Rotterdam and Marshall CT score results found an association between hospital mortality and higher CT score values [10]. In a South Korean investigation of 773 patients with TBI, Bae et al. showed that higher Rotterdam CT scores were related to in-hospital mortality [2]. Another large study in Nepal showed that higher Rotterdam and Marshall CT scores were associated with hospital mortality [11]. An investigation by Pargaonkar et al. that included 157 patients with severe TBI showed that higher Rotterdam, Helsinki, and Marshall CT scores were related to in-hospital mortality [12]. In a different South Korean investigation of 455 patients with severe TBI, Yu et al. noted that greater Rotterdam CT scores were associated with hospital death [13].

Additionally, several studies have shown that early CT scores are associated with post-hospital discharge GOS results. In the previously mentioned Bae et al. study, higher Rotterdam CT scores were found to be associated with unfavorable GOS at 12 months following hospital discharge [2]. In a study of 319 patients with TBI in Pakistan, Javeed et al. showed that Rotterdam CT scores were related to three-month GOS values [5]. In a cohort of Polish patients with TBI, Kasprowicz et al. found that Rotterdam CT scores were associated with three-month GOS values [6]. Khaki et al. found that Rotterdam, Marshall, Helsinki, and Stockholm CT scores were related to 12-month neurological function in a group of 158 Swedish patients with TBI [14]. The investigation by Yu et al. cited earlier showed that larger Rotterdam CT score values were related to post-discharge unfavorable outcomes at six months [13].

We have found only one study that combined GCS scores with CT scan results with the objective of demonstrating that their interaction effect had a better association with in-hospital mortality and post-hospital adverse outcomes than GCS sub-scores and CT scan results separately. In that investigation, the authors used specific subcomponents of the GCS and the Rotterdam score [2]. To address this gap in the literature, we aimed to assess the associations of GCS scores and prognostic CT scores separately with hospital mortality and inability to follow commands at hospital discharge and at three months following discharge. We also sought to determine if an interactive effect of GCS scores with CT scores would have a better association with these outcomes than with GCS scores or CT scores alone.

## Materials and methods

Study design and population

The patients had been admitted to Mercy Health Youngstown, a Level I trauma center, from January 27, 2012, to December 30, 2016. Inclusion criteria of this retrospective study were blunt trauma, age 18-70 years, intracranial hemorrhage with head Abbreviated Injury Scale scores of ≥2 [15], GCS score of 3-12, and mechanical ventilation for ≥five days. Exclusion criteria were as follows: penetrating trauma, intracranial hemorrhage with Abbreviated Injury Scale scores of 0 or 1, GCS score of 13-15, or mechanical ventilation for 0-4 days. The data for the current study emanates from that used in a hypertonic saline investigation [16]. In that investigation, consecutive patients meeting the inclusion criteria had been identified using the certified trauma registry data.

The CT score used in the hypertonic saline investigation was constructed to quantify the intracranial mass effect. The mass effect CT score was calculated as the sum of midline shift, lateral ventricle compression, and basal cistern compression. Each of the three findings was given a value of 0 if absent and 1 if present (theoretical range, 0-3). The presence of midline shift is also considered a risk condition for the Rotterdam and Marshall scoring systems [11,12]. One investigation reported a significant univariate association of midline shift with in-hospital mortality and post-discharge GOS results [2]. The presence of basal cistern compression is also considered a risk condition for the Rotterdam, Marshall, and Helsinki scoring systems [11,12]. An investigation specifically showed a significant univariate association of basal cistern compression with in-hospital mortality and post-discharge GOS results [2]. Lateral ventricle compression (asymmetry) has been shown to be associated with the development of a midline shift [17], failure of conservative treatment with SDH [18], and decreased brain compliance (impaired intracranial pressure control) [19].

We added an intraventricular or subarachnoid hemorrhage (SAH) component to the mass effect CT score to create a current study prognostic CT score. When SAH was present, the value was 2, whereas if it was absent, the value was 0. The theoretical range of the current study prognostic CT score was 0-5. SAH or intraventricular hemorrhages are considered risk conditions for the Rotterdam and Helsinki CT scoring systems [11,12]. Pargaonkar et al. showed that SAH is associated with early TBI mortality [12]. Bae et al. specifically demonstrated a significant univariate and multivariate association of SAH with in-hospital mortality and post-discharge GOS results [2]. Another investigation reported that six-month mortality and unfavorable outcomes were associated with SAH in univariate and multivariate analyses [20]. The current study assigned a value of 2 when SAH was present, based on the substantial effect size associations (odds ratio and risk ratio values) with adverse three-month outcomes. Other esteemed TBI researchers have also assigned a value of 2 when SAH was present and 0 when absent as a prognosticator of six-month unfavorable outcomes [20].

The GCS deficit was computed as 15 (normal GCS) minus the admission GCS value (study range, 3-12). The CT-GCS deficit score was the sum of the prognostic CT score and the GCS deficit (theoretical study range, 3-17).

Statistical analysis

Continuous data are presented as the mean ± standard deviation, whereas categorical variables are reported as frequency count and percentage. Because the mass effect score, prognostic CT score, GCS deficit, and CT-GCS deficit score are non-parametric, the Wilcoxon rank sum test was used to compare them between two independent groups. T-tests were also performed. When the p-values of the Wilcoxon and t-tests were similar, Cohen d values were computed to assess the magnitude of two intergroup mean differences. For dichotomous proportional data displayed in a 2x2 contingency table format, the two-tailed Fisher exact test was employed to assess the odds ratio and risk ratio. Multivariate logistic regression analysis was used to assess independent variable associations relative to dichotomous dependent variables. The results were entered into Excel 2010 (Microsoft Corp., Redmond, WA, USA) and imported into SAS System for Windows, release 9.2 (SAS Institute Inc., Cary, NC, USA). For receiver operating characteristic curve analyses, data were exported from SAS into MedCalc® Statistical Software, version 22.016 (MedCalc Software Ltd, Ostend, Belgium). The significance level for the p-value was set at <0.05.

## Results

Patient characteristics

A total of 112 consecutive TBI patients met the study inclusion criteria. The CT hemorrhage distribution was epidural hematoma (EDH) in eight (7.1%) patients, subdural hematoma (SDH) in 80 (71.4%), SAH in 62 (55.4%), brain contusion in 35 (31.3%), and cerebral hematoma in 25 (22.3%) patients. The mean GCS score was 6.8±3.2. The mean GCS deficit was 8.2±3.2. The GCS score and GCS deficit distributions are depicted in Table 1. The mean age was 45.3±14.2 years (18-70).

**Table 1:** GCS score and GCS deficit distribution GCS - Glasgow Coma Scale

GCS	Number	Percent	GCS deficit
3–4	38	33.9	12-11
5–8	38	33.9	10-7
9–10	14	12.5	6-5
11–12	22	19.6	4-3

The mean mass effect CT score was 2.3±0.9. The mass effect CT score distribution was as follows: none in five patients (4.5%), one in 21 (18.8%) patients, two in 23 (20.5%), and three in 63 (56.3%) patients. The mean prognostic CT score was 3.4±1.2. The prognostic CT score distribution was as follows: one in seven patients (6.3%), two in 13 (11.6%) patients, three in 49 (43.8%) patients, four in 15 (13.4%), and five in 28 (25.0%) patients. The GCS score was not associated with the prognostic CT score (p=0.1035). Among 62 patients with SAH, only five (8.1%) had no other CT intracranial hemorrhage pathology.

The mean CT-GCS deficit score was 11.6±3.6. The CT-GCS deficit score distribution is presented in Table 2.

**Table 2:** CT-GCS deficit score distribution CT - computed tomography; GCS - Glasgow Coma Scale

CT-GCS deficit score	Number	Percent
4-9	32	28.6
10-12	29	25.9
13-14	22	19.6
15-17	29	25.9

Surgical decompression

The mass effect CT score and CT-GCS deficit score had better Cohen d associations with the need for surgical decompression than the GCS score (Table 3).

**Table 3:** Surgical decompression associations GCS - Glasgow Coma Scale; CT - computed tomography Score results are mean ± standard deviation

Surgical decompression	No	Yes	Wilcoxon, p-value	T-test, p-value	Cohen d
Number	54 (48.2%)	58 (51.8%)			
GCS score	7.7 ± 3.3	6.0 ± 3.0	0.0111	0.0078	0.54
Mass effect CT score	1.7 ± 1.0	2.8 ± 0.5	<0.0001	<0.0001	1.4
CT-GCS deficit score	10.3 ± 3.4	12.8 ± 3.4	0.0003	0.0001	0.74

The proportions of surgical decompression were similar for EDH (75.0%, 6/8) and no EDH (50%, 52/104), p=0.1757. The proportion of surgical decompression was greater for SDH (61.3%, 49/80) than for no SDH (28.1%, 9/32; p=0.0013). The proportion of surgical decompression was lower for cerebral contusion/hematoma (41.5%, 22/53) than for no cerebral contusion/hematoma (61.0%, 36/59; p=0.0394). Logistic regression analyses showed that surgical decompression was associated with the mass effect score (p<0.0001) but not with SDH (p=0.3197) or no cerebral contusion/hematoma (p=0.4022). The proportion of surgical decompression was greater for the midline shift (67.9%, 55/81) than for no midline shift (9.7%, 3/31; p<0.0001; OR=19.7). The proportion of surgical decompression was greater for lateral ventricle compression (68.8%, 53/77) than for no lateral ventricle compression (14.3%, 5/35; p<0.0001; OR=13.3). The proportion of surgical decompression was greater for basal cistern compression (56.1%, 55/98) than for no basal cistern compression (21.4%, 3/14; p=0.0149; OR=4.7). Surgical decompression had independent associations with midline shift (p=0.0003) and lateral ventricle compression (p=0.0004).

EDH and SAH

EDH was not associated with hospital mortality (p=0.2523) or hospital discharge following command status (p=0.4676). The proportion of not following commands at three months was lower in those with EDH (0%, 0/7) than in those without EDH (37.9%, 39/103; p=0.0427). Logistic regression stepwise analysis showed an association for not following commands at three months with the CT-GCS deficit score (p<0.0001) but not with EDH (p>0.20). Logistic regression stepwise analysis demonstrated an association for not following commands at three months with the prognostic CT score (p=0.0040) but not with EDH (p>0.20). The proportion of SAH was greater in those not following commands at hospital discharge (67.9%, 38/56) than in those following commands (42.9%, 24/56; p=0.0081). The proportion of not following commands at three months was greater in those with SAH (47.5%, 29/61) than in those without SAH (20.4%, 10/49; p=0.0031; OR=3.5; RR=2.1).

Hospital mortality

The hospital mortality proportion was 13.4% (15/112). The GCS deficit and CT-GCS deficit scores were significantly greater in patients dying in the hospital than in the survivors (Table 4). The Cohen d for the CT-GCS deficit score was slightly larger than that for the GCS deficit.

**Table 4:** Hospital mortality associations GCS - Glasgow Coma Scale; CT - computed tomography Continuous results are mean ± standard deviation

Mortality	No	Yes	Wilcoxon, p-value	T-test, p-value	Cohen d
Number	97 (86.6%)	15 (13.4%)			
GCS score	7.1 ± 3.3	4.8 ± 2.2	0.0130	0.0091	0.82
GCS deficit	7.9 ± 3.3	10.2 ± 2.2	0.0130	0.0091	0.82
Prognostic CT score	3.4 ± 1.2	3.6 ± 1.1	0.4368	0.4619	-
CT-GCS deficit score	11.2 ± 3.6	13.8 ± 2.8	0.0111	0.0098	0.86

Not following commands at hospital discharge

The GCS deficit, prognostic CT score, and CT-GCS deficit score values were significantly greater in patients not following commands at hospital discharge than in those following commands (Table 5). The Cohen d was slightly higher for the CT-GCS deficit score than for the GCS deficit. Logistic regression stepwise analysis showed that failure to follow commands at hospital discharge was associated with the CT-GCS deficit score (p<0.0001) but not with either the GCS deficit (p>0.20) or the prognostic CT score (p>0.20).

**Table 5:** Associations with not following commands at hospital discharge GCS - Glasgow Coma Scale; CT - computed tomography Continuous results are mean ± standard deviation

Following commands	Yes	No	Wilcoxon, p-value	T-test, p-value	Cohen d
Number	56 (50%)	56 (50%)			
GCS score	8.2 ± 3.2	5.4 ± 2.6	<0.0001	<0.0001	0.96
GCS deficit	6.8 ± 3.2	9.6 ± 2.6	<0.0001	<0.0001	0.96
Prognostic CT score	3.1 ± 1.1	3.7 ± 1.2	0.0071	0.0053	0.52
CT-GCS deficit score	9.9 ± 3.2	13.3 ± 3.2	<0.0001	<0.0001	1.06

Not following commands at three months

Three-month GOS data were available for 98.2% (110/112) of the patients. The proportion following commands at three months (64.5%) was greater than the proportion following commands at hospital discharge (50.0%; p=0.0308; OR=1.8). The proportion of not following commands at three months was similar with and without SDH (p=0.4678), cerebral contusion (p=0.8004), and cerebral hematoma (p=0.8127). The GCS deficit, prognostic CT score, and CT-GCS deficit score values were greater in patients not following commands at three months than in those following commands (Table 6). The Cohen d was greater for the CT-GCS deficit score than for the GCS score and GCS deficit.

**Table 6:** Associations with not following commands at three months (continuous data) GCS - Glasgow Coma Scale; CT - computed tomography Continuous results are mean ± standard deviation

Following commands	Yes	No	p-value	Cohen d
Number	71 (64.5%)	39 (35.5%)		
GCS score	7.6±3.2	5.3±2.7	0.0002	0.78
GCS deficit	7.4±3.2	9.7±2.8	0.0002	0.78
GCS deficit median (interquartile range)	8.0 (4-10)	11.0 (8-12)	0.0004	
Prognostic CT score	3.2±1.1	3.9±1.1	0.0028	0.64
Prognostic CT score median (interquartile range)	3.0 (3-4)	4.0 (3-5)	0.0033	
CT-GCS deficit score	10.5±3.4	13.6±3.1	<0.0001	0.94
CT-GCS deficit score median (interquartile range)	11.0 (8-13)	14.0 (12-16)	<0.0001	

Not following commands at three months was independently associated with the GCS score (p=0.0013) and the prognostic CT score (p=0.0120). Logistic regression stepwise analysis showed that the failure to follow commands at three months was associated with the CT-GCS deficit score (p<0.0001) but not with either the GCS deficit (p>0.20) or the prognostic CT score (p>0.20).

Results were dichotomized to show three-month outcome associations with the GCS deficit (Table 7) and the CT-GCS deficit score (Table 8). For the two GCS deficit and the two CT-GCS deficit score cohorts, the mean GCS scores were virtually identical for the two lower-value groups (9.5 and 9.2) and for the two higher-value groups (3.9 and 3.9). For the CT-GCS deficit scores, the odds ratio and risk ratio values were higher, but the p-value was lower for not following commands at three months when compared with those for the GCS deficit scores (Tables 7 and 8). The areas under the receiver operating characteristic curve (AUC) for no commands at three months were 0.70 (p<0.001) for the GCS score and 0.75 (p<0.001) for the CT-GCS deficit score.

**Table 7:** Association for not following commands at three months according to GCS deficit scores 3-8 versus 9-12 GCS - Glasgow Coma Scale GCS score results are mean ± standard deviation

GCS deficit score	3-8	9-12	p-value	Odds ratio	Risk ratio
GCS score	9.5±2.0	3.9±1.1			
Number	57 (51.8%)	53 (48.2%)			
No commands	12 (21.1%)	27 (50.9%)	0.0014	3.9	2.1

**Table 8:** Association for not following commands at three months according to CT-GCS deficit scores 4-12 versus 13-17 CT - computed tomography; GCS - Glasgow Coma Scale GCS score results are mean ± standard deviation

CT-GCS deficit score	4-12	13-17	p-value	Odds ratio	Risk ratio
GCS score	9.2±2.2	3.9±1.2			
Number	60 (54.5%)	50 (45.5%)			
No commands	11 (18.3%)	28 (56.0%)	0.0001	5.7	3.1

## Discussion

The inclusion criteria of the current study required the presence of intracranial hemorrhage, which is prima facie evidence for TBI. The exclusion of patients with GCS scores of 12-15 eliminated those with minor TBI. The requirement for ≥five days of mechanical ventilation excluded those dying of devastating TBI in the first four hospital days. These criteria portend a balance between TBI homogeneity and heterogeneity that we believe likely fosters the ability to identify proximal TBI conditions that can better be associated with distal TBI outcomes. This is in contradistinction to including the whole spectrum of TBI that can create noise that mitigates the ability to identify proximal TBI conditions as they relate to distal TBI outcomes. However, excessive inclusion criteria might create misrepresentative associations that lack erudition.

GCS score and GCS deficit associations

Most of the patients in the current study had GCS scores of 3-8. The GCS deficit in the current study had a large association (Cohen d) with in-hospital mortality. A similar GCS relationship with hospital mortality has also been found by other investigators [1-3]. The current study demonstrated that the admission GCS score has a large association with not following commands at hospital discharge. Another investigation also showed that hospital GOS results were associated with admission GCS scores [21]. The GCS score has also been found to have an association with the Rotterdam score, in general, and midline shift, in particular [7]. The current study also showed a large association (Cohen d) between the GCS score and not following commands at three months. A similar relationship between the GCS score and post-hospital discharge GOS results was demonstrated by several other researchers [1-7].

These findings from the current study and literature suggest that the observed investigational GCS values were credible. The study findings and literature coherency also suggest that the exclusion of the clinical extremes of patients with TBI did not distort the importance of the GCS score in the current investigation. The GCS deficit was created to convert lower GCS values, which imply worse neurological function, into positive expressions. We thought that this would make a more aesthetic representation for interacting with positive or worse prognostic CT scores. Of relevance, another TBI investigation has inverted GCS motor scores for the same purpose [20].

Mass effect CT score

The CT score used in the hypertonic saline investigation was constructed to quantify the intracranial mass effect [16]. In-kind, the current study's mass effect CT score also considered the presence or absence of midline shift, basal cistern compression, and lateral ventricle compression. In the current study, the need for surgical decompression had univariate associations with midline shift, basal cistern compression, and lateral ventricle compression. Although lateral ventricle compression (asymmetry) has not been widely reported as a CT risk condition, the need for surgical decompression had independent associations with midline shift and lateral ventricle compression. Ostensibly, the validity of the mass effect CT schema lies in the strong association (Cohen d) with the need for surgical decompression. Other investigators have provided evidence that midline shift and basal cistern compression are associated with surgical decompression in TBI [22].

Prognostic CT score

We created a prognostic CT score by adding SAH (no or yes) to the mass effect CT score. SAH was found to have an association with not following commands at hospital discharge and at the three-month follow-up. It is relevant that the three-month outcome univariate odds ratio and risk ratio values for SAH in the current study were similar to those in the six-month unfavorable outcomes investigation [20].

We found that EDH had no association with either hospital mortality or following commands at hospital discharge. Although a lower proportion of patients with EDH were not following commands at three months than those without EDH, this finding became insignificant when also considering the prognostic CT score results. The construction of the Rotterdam and Helsinki scoring systems considers the presence of EDH as a decreased risk condition for adverse clinical outcomes [12]. A large analysis by Steyerberg et al. specifically found that EDH was a decreased risk condition in univariate analysis for unfavorable outcomes at six months [20]. Another investigation found that EDH had a univariate association with decreased six-month unfavorable outcomes; however, the effect size was weak (risk ratio of 0.83), indicating little to no decrease in risk [13]. In univariate analysis, Bae et al. found that EDH was a decreased risk condition (odds ratio of 0.48) for 12-month post-discharge unfavorable outcomes; however, they found an increased risk in multivariate analysis (odds ratio of 4.3) [2]. Thus, evidence exists to demonstrate that EDH has a decreased risk, a minimal to no decreased risk, and an increased risk for post-hospital discharge adverse outcomes. Owing to these conflicting literature observations and the current study findings, we recommend that EDH not be a routine stratification component for TBI prognostic brain CT scoring systems.

The prognostic CT score was higher in patients not following commands at hospital discharge than in those following commands; however, the Cohen d value of the GCS score was larger. Numerous researchers have shown that early CT score findings have associations with in-hospital mortality [2,8-13]. Unfortunately, we have been unable to readily identify any publication describing GOS results at the time of hospital discharge in relationship to early CT score results. The prognostic CT score was also higher in patients not following commands at three months than in those following commands; yet, the Cohen d value of the GCS score was greater. Several research investigations have shown that early CT score results are associated with post-hospital discharge GOS results [2,5,6,13,14].

Prognostic CT score and GCS deficit interaction associations

The CT-GCS deficit score was the sum of the prognostic CT score and the GCS deficit. Because the GCS deficit was not associated with the prognostic CT score, it becomes plausible that the two entities could create an interactive effect regarding outcomes. The CT-GCS deficit score was higher in patients with in-hospital mortality than in the survivors. The Cohen d value of the CT-GCS deficit score was slightly greater for in-hospital mortality than that for the GCS deficit. Although the prognostic CT score was not different for those dying, the slightly larger Cohen d value of the CT-GCS deficit score suggests that the prognostic CT score and GCS deficit had an interactive effect.

The CT-GCS deficit score was higher in patients not following commands at hospital discharge than in those following commands. For patients not following commands at hospital discharge, the Cohen d value of the CT-GCS deficit score was slightly higher than that for the GCS deficit, implying that the prognostic CT score and GCS deficit had an interactive result. This observation is further supported by the fact that during the stepwise multivariate logistic regression analysis for no commands at hospital discharge, the CT-GCS deficit score was selected, yet the prognostic CT score and GCS deficit were excluded. These data indicate that a mass effect and SAH composite CT score can interact with the GCS score to better prognosticate TBI outcomes than the GCS score alone.

The CT-GCS deficit score was higher in patients not following commands at three months than in those following commands. For those not following commands at three months, the Cohen d value of the CT-GCS deficit score was substantially greater than that for the GCS deficit. This observation is compelling for an interactive effect between the prognostic CT score and GCS deficit. Multivariate logistic regression analysis corroborated this impression in that it demonstrated not following commands at three months was independently associated with the prognostic CT score and GCS deficit. This advantage of the prognostic CT score and GCS deficit interaction was further depicted in that during stepwise multivariate logistic regression analysis for no commands at three months, the CT-GCS deficit score was selected, yet the prognostic CT score and GCS deficit were excluded. The proportion of those not following commands at three months was greater among those with a GCS deficit of 9-12 than among those with a GCS deficit of 3-8. Similarly, the proportion of those not following commands at three months was greater among those with a CT-GCS deficit score of 13-17 than in those with a CT-GCS deficit score of 4-12. It is important to note that the mean GCS score was similar for the lower GCS deficit and CT-GCS deficit score groups. Likewise, the mean GCS score was similar for the higher GCS deficit and CT-GCS deficit score groups. These observations imply that the comparisons of the GCS deficit and the CT-GCS deficit score occurred in similar patients.

When comparing the GCS deficit and the CT-GCS deficit score results, the p-value was lower, and the odds ratio and risk ratio values were higher for the CT-GCS deficit score results. This indicates that the CT-GCS deficit score, which combines the prognostic CT score and GCS deficit, has a greater association with no commands at three months than the GCS score alone. The AUC for the CT-GCS deficit score was slightly higher for discriminating patients not following commands at three months when compared to the GCS deficit. This also suggests that the prognostic CT score and GCS deficit have an interactive effect. Because the proportion of patients following commands at three months was significantly greater than that at hospital discharge, it may be important to assess potential post-discharge outcomes on the basis of interactive effects of the prognostic CT score and GCS deficit. Finally, in TBI prospective trials and retrospective analyses, there may be value in displaying intergroup similarities or heterogeneities using the GCS score, prognostic CT score, and CT-GCS deficit score. In summary, the data indicate that a mass effect and SAH composite CT score can interact with the GCS score to better prognosticate TBI outcomes than the GCS score alone.

The authors have several observations regarding an investigation that interacted GCS score and Rotterdam CT score subcomponents [2]. Importantly, the study included the extremes of TBI (GCS scores of 3-8 and 13-15) with marked distinctions. The very low mortality for the GCS scores of 13-15 markedly contrasts with the quite high mortality for GCS scores of 3-8 (risk ratio of 337). Likewise, the very low unfavorable outcome for GCS scores of 13-15 is substantially distinct from the quite high unfavorable outcome for GCS scores of 3-8 (risk ratio of 238). From Table 1 in the manuscript, we computed the mean Rotterdam scores to be 2 for patients with GCS scores of 13-15 patients and 4 for patients with GCS scores of 3-8 patients. The AUCs for the GCS score, Rotterdam CT score, and new TBI score were nearly 1 for in-hospital mortality and post-discharge unfavorable outcomes. Such a large AUC is astonishing and atypical for most clinical investigations. This near-perfect sensitivity-specificity discrimination seems likely to be related to the inclusion of the extremes of patients with TBI. That is, the inclusion of GCS scores of 13-15, the majority of patients, and all GCS scores of 3-8, including early deaths with devastating TBI, may overstate evident group traits or over-look more subtle intra-group variances that are clinically important to those managing patients with TBI. Although the AUCs for not following commands at three months in the current study are less than the Bae et al. investigation [2], it may be, in part, related to the exclusion of the extremes of TBI severity. We are perplexed as to how EDH could be found to be a decreased univariate risk condition for in-hospital mortality and post-discharge unfavorable outcomes but an increased risk condition when conducting multivariate analyses [2]. The current authors have neither an intuitive, rational, nor clinical reason for this statistical paradox. Further, the AUC for EDH relative to in-hospital mortality and unfavorable outcomes was <0.60 [2]. Although the Bae et al. investigation [2] used the GCS components, verbal and motor, to include in their new TBI score, other investigators have shown that the total GCS score is more sensitive than the motor score for identifying trauma patients with serious injury [23]. Despite some perplexities, we commend Bae et al. for advancing the notion that TBI GCS and brain CT findings are likely to have an interactive effect relative to clinical outcomes.

The prognostication components of the conjoint GCS and brain CT findings used in Bae et al.'s study, and the current study have several distinctions and one similarity. Bae et al.'s study included GCS subcomponents (verbal and motor), whereas the current study used the total GCS score (GCS deficit). Bae et al. study included an EDH component, whereas the current study excluded consideration of EDH presence or absence. Additionally, the Bae et al. study excluded a mass effect component (midline shift and basal cistern compression), whereas the current study included mass effect elements (midline shift, basal cistern compression, and lateral ventricle compression). However, both investigations included an SAH component. A comparison of the two conjoint GCS and brain CT methods would require the computation of a total score for each in the same TBI cohort and their associations with adverse outcomes.

Limitations of the study

The main limitation of the current study is that it was a retrospective analysis. The study findings need to be confirmed in a prospective observational investigation. Preferably, the investigation would include a larger sample size and possibly a study design with less restrictive inclusion criteria.

## Conclusions

The mass effect CT score had a substantially better association with the need for surgical decompression than did the GCS score. The associations for not following commands at hospital discharge were greater with the CT-GCS deficit score than with the GCS deficit. The associations for not following commands at three months were also greater with the CT-GCS deficit score than with the GCS deficit. These observations support the notion that a mass effect and SAH composite CT score can interact with the GCS score to better prognosticate TBI outcomes than the GCS score alone. These findings need corroboration in a larger cohort during prospective observation. Owing to inconsistent literature observations and the current study findings, we recommend that EDH not be a routine stratification component for TBI brain CT scoring systems.
